# Supplementary material for: Establishment of the Embryonic Shoot Meristem Involves Activation of Two Classes of Genes with Opposing Functions for Meristem Activities
Source: Int J Mol Sci. 2020 Aug 15;21(16):5864. doi: 10.3390/ijms21165864 (PMC7461597; doi:10.3390/ijms21165864)
Supplement: Supplementary file 1 [file ijms-21-05864-s001.zip › Supplementary_figures_revised.pdf]

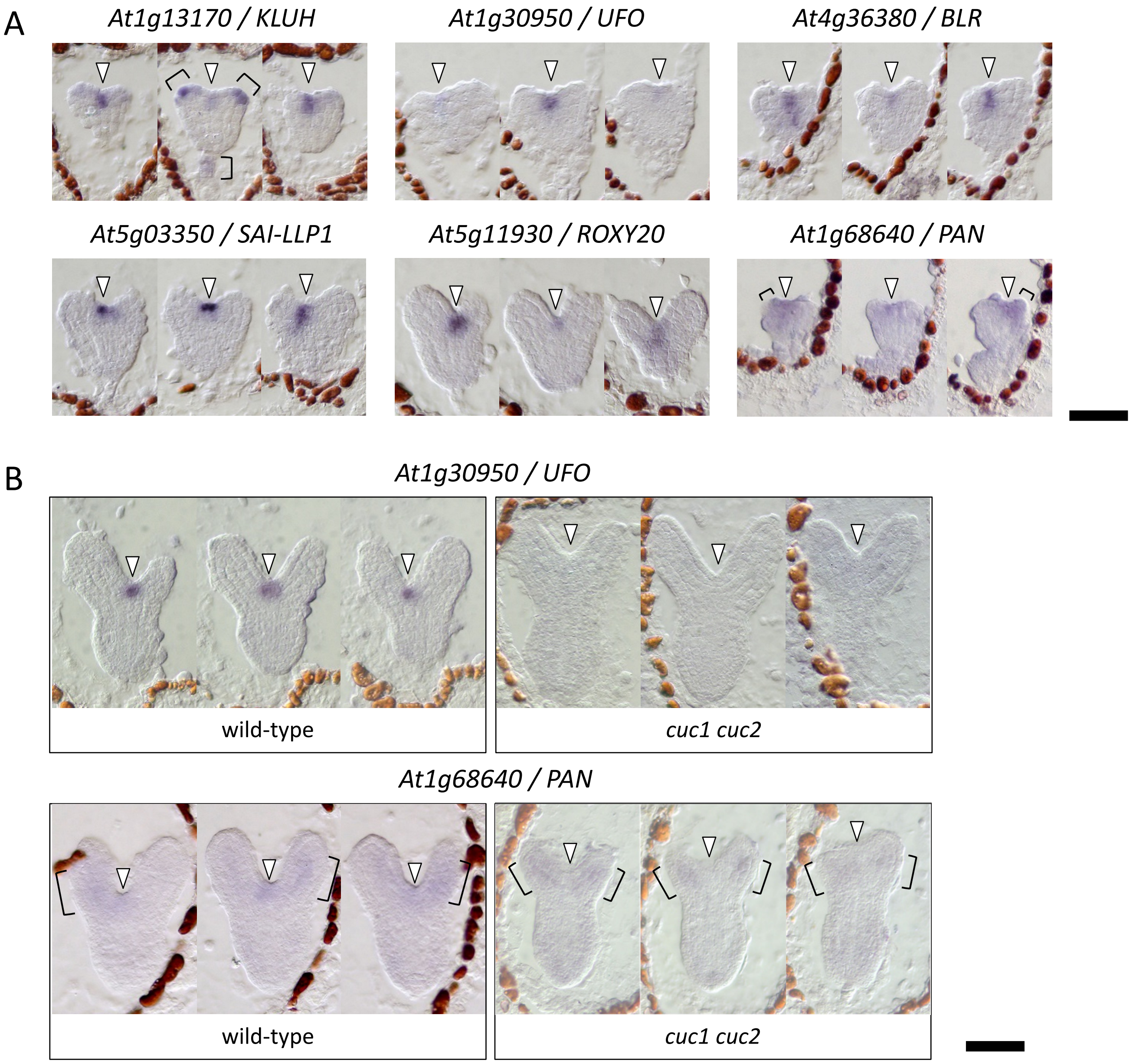

Figure S1. Expression of candidate downstream genes in wild-type L*er* and *cuc1-1 cuc2-1* embryos. (A) *In situ* hybridization of the six newly identified candidate downstream genes in wild-type embryos at the early- to mid-heart stages. For each gene, three serial longitudinal are shown. (B) *In situ* hybridization of the two candidate genes that are not included in Figure 1. Three serial longitudinal sections of wild-type L*er* (left) and *cuc1-1 cuc2-1* double-mutant (right) embryos at the late heart stage. Arrowheads indicate the position of the cotyledon boundary region and brackets indicate the position of expression outside the boundary region. Scale bars, 50 μm.

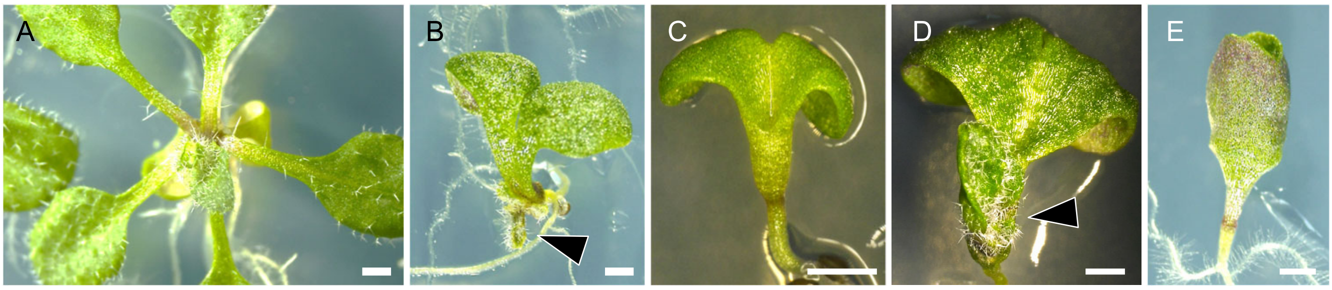

Figure S2. Examples of rescued and non-rescued plants. (A and B) Seedlings of the *cuc1-5 cuc2-3* double homozygote transformed with the *CUC2* transgene as a positive control at 14 dag with stongly (A) or mildly (B) rescued phenotypes. (C and D) A seedling of the *cuc1-5 cuc2-3* double homozygote with the *LAS* transgene at 6 dag (C) and at 21 dag (D), showing a mildly rescued phenotype. (E) Non-rescued *cuc1-5 cuc2-3* homozygous seedling transformed with the *ROXY20* transgene at 12 dag. Arrowheads in (B) and (D) indicate delayed shoots. Bars, 1 mm.
