# Supplementary material for: Establishment of the Embryonic Shoot Meristem Involves Activation of Two Classes of Genes with Opposing Functions for Meristem Activities
Source: Int J Mol Sci. 2020 Aug 15;21(16):5864. doi: 10.3390/ijms21165864 (PMC7461597; doi:10.3390/ijms21165864)
Supplement: Supplementary file 1 [file ijms-21-05864-s001.zip › Supplementary_text_S1.docx]

**Text S1**

**Screening of additional genes regulated by *CUC1* and *CUC2***

To identify genes whose expression requires *CUC1* and *CUC2* during embryogenesis, we first carried out transcriptome analysis that compared gene expression in the wild type (Landsberg *erecta*) and *cuc1-1 cuc2-1* double mutant embryos. Based on two hybridization experiments using the same set of RNA samples with dye-swapped labeling, we identified 52 genes whose expression was significantly downregulated in *cuc1 cuc2* double mutant with P values less than 0.01 in both experiments (Table S1). One of these genes was *STM* (*At1g62360*), which had already been reported to be downregulate in *cuc1 cuc2* [1] and the remaining 51 were not previously analyzed their expression in *cuc1 cuc2*.

We then carried out quantitative reverse transcription polymerase chain reaction (qRT-PCR) experiments for these 51 genes, using RNA extracted from heart and bending cotyledon stage embryos of each genotype (Table S2,S3). We selected nine genes consisting of At1g30950, At1g68640, At5g0335 and At5g11930, which were downregulated in the mutant specifically at the heart stage, and *At1g13710*, *At3g53230*, *At4g04610*, *At4g21990* and *At5g03210*, which were downregulated specifically at the bending cotyledon stage. *STM*, which we used as a positive control, was downregulated at both stages.

Finally, these candidate downstream genes were subjected to *in situ* hybridization experiments to localize their expression in wild type and *cuc1 cuc2* embryos. In addition to the above nine genes, we also added another gene, *BELLRINGER* (*BLR*, *At5g02030*), to a candidate because it has been identified as a gene activated in *35S-CUC1* seedlings in our previous microarray experiments [2] and its function in shoot development has been reported [3-5]. Among the ten candidates, two showed ubiquitous expression (At3g53230 and At5g03210) and two showed no detectable expression in wild type embryos (At4g04610 and At4g21990). These genes were therefore excluded for further analysis. Expression of the remining six genes was detected in the cotyledon boundary region (Fig 1A to D; Supplementary Fig S1A, S1B), where *CUC1* and *CUC2* are expressed [1,6]. These genes were *KLUH/CYP78A5* (*At1g13710*), *UNUSUAL FLORAL ORGANS* (*UFO*, *At1g30950*), *PERIANTHIA* (*PAN*, *At1g68640*), *BLR*, *SA-INDUCED LEGUME LECTIN-LIKE PROTEIN 1* (*SAI-LLP1*, *At5g03350*) and *ROXY20* (*At5g11930*). Two of them (*UFO* and *SAI-LLP1*) were expressed exclusively in the boundary region whereas the rest were expressed also in other regions such as cotyledon primordia (*KLUH* and *PAN*), hypocotyl (*BLR*) and root tip (*ROXY20*). In *cuc1 cuc2* embryos, expression of all six genes was missing specifically in the boundary region, suggesting largely cell-autonomous regulation of these genes by *CUC1* and *CUC2*. These results showed that expression of the six genes in the boundary region is dependent on *CUC1* and *CUC2*.

**Materials and Methods**

~100 embryos of Landsberg *erecta* (L*er*) and *cuc1-1 cuc2-1* were excised in 6% glucose and fixed overnight in ethanol:acetic acid (3:1) at 4°C. Fixed embryos were washed with ethanol and total RNA was isolated using RNeasy Plant Mini Kit (QIAGEN) according to manufacturer’s instruction. Isolated RNA samples were subjected to two-color Arabidopsis 2 Oligo Microarray (22K, Agilent technologies). Generation of cRNA probe using Low RNA Fluorescent Linear Amplification Kit (Agilent Technologies), hybridization and data processing were carried out by Hokkaido System Science Co., Ltd. Genes downregulated in *cuc1 cuc2* with PValueLogRatio <0.01 [7] in both experiments were selected. The data discussed in this publication have been deposited in NCBI's Gene Expression Omnibus [8] and are accessible through GEO Series accession number GSE 147149 (https://www.ncbi.nlm.nih.gov/geo/query/acc.cgi?acc=GSE 147149). qRT-PCR experiments were carried out as previously reported [2].

**References**

1. Aida, M.; Ishida, T.; Tasaka, M. Shoot apical meristem and cotyledon formation during *Arabidopsis* embryogenesis: interaction among the *CUP-SHAPED COTYLEDON* and *SHOOT MERISTEMLESS* genes. *Development* **1999**, *126*, 1563-1570.

2. Takeda, S.; Hanano, K.; Kariya, A.; Shimizu, S.; Zhao, L.; Matsui, M.; Tasaka, M.; Aida, M. CUP-SHAPED COTYLEDON1 transcription factor activates the expression of *LSH4* and *LSH3*, two members of the ALOG gene family, in shoot organ boundary cells. *Plant J* **2011**, *66*, 1066-1077, doi:10.1111/j.1365-313X.2011.04571.x.

3. Byrne, M.E.; Groover, A.T.; Fontana, J.R.; Martienssen, R.A. Phyllotactic pattern and stem cell fate are determined by the *Arabidopsis* homeobox gene *BELLRINGER*. *Development* **2003**, *130*, 3941-3950, doi:10.1242/dev.00620.

4. Roeder, A.H.; Ferrándiz, C.; Yanofsky, M.F. The role of the REPLUMLESS homeodomain protein in patterning the Arabidopsis fruit. *Curr Biol* **2003**, *13*, 1630-1635, doi:10.1016/j.cub.2003.08.027.

5. Smith, H.M.; Hake, S. The interaction of two homeobox genes, *BREVIPEDICELLUS* and *PENNYWISE*, regulates internode patterning in the Arabidopsis inflorescence. *Plant Cell* **2003**, *15*, 1717-1727, doi:10.1105/tpc.012856.

6. Takada, S.; Hibara, K.; Ishida, T.; Tasaka, M. The *CUP-SHAPED COTYLEDON1* gene of *Arabidopsis* regulates shoot apical meristem formation. *Development* **2001**, *128*, 1127-1135.

7. Zahurak, M.; Parmigiani, G.; Yu, W.; Scharpf, R.B.; Berman, D.; Schaeffer, E.; Shabbeer, S.; Cope, L. Pre-processing Agilent microarray data. *BMC Bioinformatics* **2007**, *8*, 142, doi:10.1186/1471-2105-8-142.

8. Edgar, R.; Domrachev, M.; Lash, A.E. Gene Expression Omnibus: NCBI gene expression and hybridization array data repository. *Nucleic Acids Res* **2002**, *30*, 207-210, doi:10.1093/nar/30.1.207.
